# Impact of Burdizzo and Surgical Castration on Immune and Oxidative Stress Markers in Cattle

**DOI:** 10.3390/vetsci12060537

**Published:** 2025-06-01

**Authors:** Thanyakorn Chalalai, Piyarat Srinontong, Worapol Aengwanich, Kanticha Srisila, Sudarat Promkrathok, Mookdawan Sununta, Bhuripit Saraphol, Zhiliang Wu

**Affiliations:** 1 Faculty of Veterinary Sciences, Mahasarakham University, Mahasarakham 44000, Thailand; thanyakorn.c@msu.ac.th (T.C.); worapol.a@msu.ac.th (W.A.); 62012210002@msu.ac.th (K.S.); 62012210023@msu.ac.th (S.P.); 62012210038@msu.ac.th (M.S.); bhuripit.s@msu.ac.th (B.S.); 2 Bioveterinary Research Unit, Mahasarakham University, Mahasarakham 44000, Thailand; 3 Stress and Oxidative Stress in Animal Research Unit, Faculty of Veterinary Sciences, Mahasarakham University, Mahasarakham 44000, Thailand; 4 Department of Parasitology and Infectious Diseases, Gifu University Graduate School of Medicine, Gifu 5011194, Japan; wu.zhiliang.t7@f.gifu-u.ac.jp

**Keywords:** castration, cattle, cytokines, inflammation, oxidative stress

## Abstract

Castration is an essential procedure in beef cattle management, designed to enhance meat quality, regulate aggressiveness, and control undesirable breeding. This study compared the impacts of Burdizzo and surgical castration on the immunological, oxidative, and inflammatory responses in Angus calves. Calves were randomly assigned to the Burdizzo (n = 4) and surgical (n = 4) castration groups. Blood samples were collected before castration and 3, 6, 24, and 48 h post-castration for the measurement of the pro-inflammatory cytokines TNF-α and IFN-γ, the anti-inflammatory cytokine IL-10, the oxidative stress markers malondialdehyde and nitric oxide, the total antioxidant capacity, and peripheral blood mononuclear cell populations, including CD3^+^CD4^+^, CD3^+^CD8^+^, and CD3^+^WC1^+^ T-cells. The results showed that Burdizzo castration caused significantly higher levels of IFN-γ at 3 and 6 h, TNF-α at 24 and 48 h, IL-10 at 3 and 24 h, and malondialdehyde at 24 h post-castration (*p* < 0.05), indicating more intense inflammatory and oxidative stress responses. Both methods caused alterations in CD3^+^CD4^+^ and CD3^+^CD8^+^ T-cell counts, while Burdizzo castration induced longer-lasting immune activation. These findings indicate that surgical castration may provide welfare advantages by mitigating prolonged inflammation and stress and promoting faster recovery. This study offers insights into optimal castration practices prioritizing productivity and animal welfare.

## Simple Summary

Castration is a routine management practice in livestock, but its impact on inflammation and stress responses varies depending on the method used. This study compares Burdizzo and surgical castration in cattle by evaluating inflammatory cytokines, oxidative stress markers, and the immunological response, including immune cell subpopulations. The results indicate that Burdizzo castration induced a more severe initial inflammatory response and caused long-term immune activation. These findings provide a better understanding of the physiological response following castration, providing valuable insights for improving animal welfare and pain management strategies.

## 1. Introduction

Beef cattle farming plays an important role in economic growth and rural livelihoods. In developed countries, commercial agriculture contributes to the total agricultural gross domestic product (GDP). In contrast, in developing countries, beef cattle farms provide an important livelihood source for farmers, supporting employment, household economies, and diets and nutrition for rural communities [1,2,3]. Global meat consumption of all categories has increased over the years to support the fast-growing urban populations and the rising rural incomes. This has led to expanding intensive and extensive cattle farming systems, making effective herd management crucial for reproductive productivity, farm animal welfare, and profitability [4]. The advancement of agricultural techniques via invention and innovation is necessary to improve production for sustainable development [5,6].

In beef cattle farming, castration is a critical management practice intended to enhance meat quality, reduce aggressive behavior, and prevent unwanted reproduction [6,7]. Generally, both Burdizzo and surgical castration are employed in beef cattle production, but their usage varies by region, production system, and animal age [8]. The Burdizzo method is a bloodless procedure that does not involve cutting the skin, which lowers the risk of infection and blood loss, especially in young calves [9]. In commercial operations, surgical castration is more widely practiced due to its effectiveness in ensuring the complete removal of testicular tissue, especially in older calves. However, surgical castration poses a higher risk of bleeding and subsequent infections [10]. In these castration procedures, pain management is crucial from the perspectives of animal welfare and productivity, and the efficacy of each technique and its implications for welfare are constantly being assessed [11].

Castration is a routine cattle management procedure, though it imposes notable physiological and biological responses in addition to behavioral effects. Although Burdizzo and surgical castration methods induce acute pain, the level and duration differ; for example, the surgical method typically causes longer-lasting discomfort [10]. The physiological changes associated with pain directly stimulate the release of hormones from the hypothalamic–pituitary–adrenal axis, particularly cortisol, and stress-related hormones [12,13]. This activation not only affects immediate physiological responses but also has longer-term consequences for animal welfare and productivity [14]. The release of pro-inflammatory cytokines like TNF-α and IFN-γ, as well as anti-inflammatory cytokines like IL-10, which serve to decrease inflammatory effects and initiate tissue repair, is another characteristic of the inflammatory response that occurs after castration [15]. Previous studies have demonstrated that the first 48 to 72 h following castration represents a critical window for observing acute-phase immune and physiological responses. During this period, key inflammatory markers and stress indicators tend to peak, providing a reliable timeframe for evaluating the early biological impact of castration [16,17,18]. Therefore, our study focused on this acute recovery phase to capture the short-term immune and oxidative stress responses.

Excessive inflammatory and stress responses are detrimental to the welfare and productivity of cattle. Elevated inflammation, marked by increased cytokine release, delays wound healing and negatively affects overall health, as highlighted in several recent studies [6,15,19]. Prolonged inflammation exacerbates tissue damage and compromises immune function, which increases oxidative stress [16]. Studies have indicated that cytokine storms induced by excessive inflammation can lead to severe systemic effects, including reduced feed intake, weight loss, and increased susceptibility to secondary infections [18,20]. These adverse effects emphasize the importance of developing castration protocols that minimize inflammatory responses while ensuring effective outcomes.

Cattle castration causes oxidative stress, a vital physiological response characterized by an imbalance between antioxidant defense and reactive oxygen species (ROS) [21]. Malondialdehyde (MDA), a biomarker for lipid peroxidation, is a key indicator of this oxidative stress and is frequently used to evaluate stress levels after castration. Studies indicate that both Burdizzo and surgical castration causes high MDA levels, suggesting that calves undergoing either procedure suffer from severe oxidative stress [22,23]. Additionally, castration may disrupt the generation of nitric oxide (NO), which is essential for numerous physiological functions. This can cause oxidative stress and trigger inflammation in the injured tissues [24].

Castration induces alterations in immune cell subpopulations, particularly lymphocytes, such as CD3^+^CD4^+^ T-helper cells and CD3^+^CD8^+^ cytotoxic T cells, which play a key role in regulating immune responses to tissue damage [20]. Although both Burdizzo and surgical castration induce immune responses, Burdizzo castration induces a delayed response, possibly due to its less invasive nature [6]. Nonetheless, Burdizzo castration can still trigger a robust systemic inflammatory response because of the ischemic damage caused by the crushing of spermatic cords [15].

Although previous studies have revealed the physiological effects of castration, gaps remain regarding the comparative impacts of Burdizzo and surgical castration, especially concerning the timing and intensity of stress, oxidative stress, and immune responses. The relationship between cytokine expression, oxidative stress, and the dynamic changes in immune cells, especially lymphocytes after castration, is still poorly understood. Therefore, the aim of this study is to compare Burdizzo and surgical castration in terms of stress, oxidative stress, antioxidant status, cytokine expression, and immune cell populations in beef calves. To better understand the short-term responses to castration in beef calves, the changes in these markers were analyzed at different time points (before castration and 3, 6, 24, and 48 h post-castration). This study is expected to reveal significant differences between the two methods, particularly in how they impact animal welfare and stress responses. This study will provide basic knowledge of the best practices for castration, helping to reduce pain and stress in cattle. Furthermore, the results may assist in developing more effective analgesic protocols tailored to the specific needs of each castration method.

## 2. Materials and Methods

### 2.1. Animal Housing and Management

The animal study protocol was approved by the Institutional Ethics Committee on Animal Experimentation at Mahasarakham University (IACUC-MSU-7/2024, 13 February 2024).

Eight male Angus calves, aged between 5 and 8 months and weighing 100–160 kg, were selected for this study, conducted from February to June 2024. All calves were housed on a farm in Mahasarakham, northeastern Thailand. A veterinarian performed physical exams on the calves before the experiment to verify their health. The calves received water ad libitum, concentrated feed, and were allowed to graze freely. All animals were vaccinated with a trivalent foot-and-mouth disease vaccine (O, A, and Asia1 serotypes) and dewormed every four months.

### 2.2. Experimental Procedure

The calves were randomly assigned into two groups (n = 4 per group), designated as the Burdizzo castration and surgical castration groups. The sample size for castrated cattle was determined by Ilyas et al. [25].

All calves received an intramuscular injection of xylazine hydrochloride (X-Lazine^®^ 20 mg: L.B.S. Laboratory LTD., Bangkok, Thailand) at 0.02 mg/kg body weight 20 min before the castration procedures. During the procedures, the calves were restrained gently using a halter.

For both groups, the scrotum was cleaned with alcohol and a povidone–iodine solution. During the Burdizzo castration procedure, as described by Fisher et al. [12], each spermatic cord was crushed for two 10 s periods by a Burdizzo clamp around the neck of the scrotum, being careful not to overlap the crushed cords. Surgical castration was assessed using an open technique, as reviewed by Marquette et al. [10]; a horizontal incision was made with a scalpel through approximately the distal third of the scrotal wall and subcutaneous tissue to expose the testicles. The testes were separated from their attachments. Two sets of hemostats were used to make a crush line to place an encircling ligature. The testes were then removed using the encircling ligature and by cutting the tunica vaginalis and cord with a scalpel below a second ligature. The surgical wound was left open to promote healing and enhance drainage. After the procedure, a povidone–iodine solution was applied to prevent infection.

### 2.3. Blood Collection

Blood samples (20 mL from each calve) from eight male Angus calves were collected from the jugular vein immediately before castration and 3, 6, 24, and 48 h post-castration in tubes without anticoagulant (Meditube^®^ clot activator, Hebei Xinle Sci & Tech Co., Ltd., Xinle, China), centrifuged (Hettich Rotina 380R, Andreas Hettich GmbH & Co. KG, Tuttlingen Germany) at 3000× *g* at 4 °C for 15 min, transferred into clean tubes, and stored at −20 °C until serum analysis. For peripheral blood mononuclear cell (PBMC) isolation, blood was collected in EDTA-vacutainer tubes (Meditube^®^ K3EDTA, Hebei Xinle Sci & Tech Co., Ltd., Xinle, China).

### 2.4. Peripheral Blood Mononuclear Cell Isolation

Blood samples were obtained from EDTA-vacutainer tubes and diluted with phosphate-buffered saline (PBS) (Vivantis, Selangor Ehsan, Malaysia) and underwent density gradient centrifugation on Ficoll-Paque Plus (Cytiva™, MA, USA) in 15 mL centrifuge tubes and centrifuged at 800× *g* for 30 min. PBMCs, located in the interphase of the obtained layers, were transferred into a fresh 15 mL centrifuge tube. Cells were washed twice with PBS. Erythrocytes were lysed with RBC lysis buffer (Sigma-Aldrich, MO, USA), and the cells were washed with PBS, centrifuged at 1400× *g* for 10 min, and suspended with PBS containing 2% fetal bovine serum (Invitrogen, CA, USA) for flow cytometry analysis or stored in RNAlater^®^ solution (Invitrogen, CA, USA) for RNA isolation.

### 2.5. Flow Cytometry Analysis

**Figure 1:**
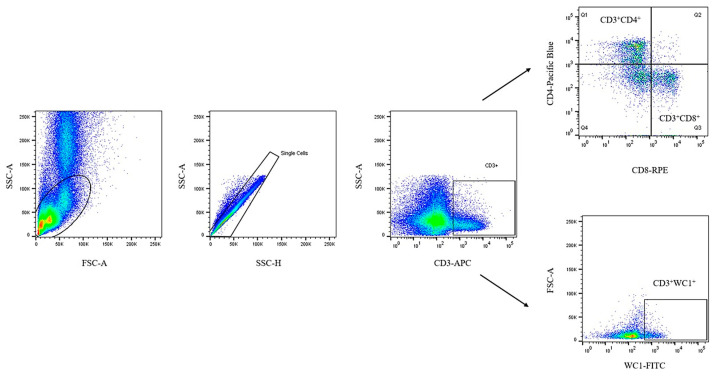
Gating strategy for the flow cytometry analysis to bovine T cells and subpopulations in PBMCs. Cells were labeled with anti-CD3 Alexa Fluor 647, anti-CD4 Pacific Blue, anti-CD8 RPE, and anti-WC1 FITC.

The PBMCs were stained with polyclonal rat IgG (Sigma-Aldrich, MO, USA) at 4 °C for 15 min, then stained with the following antibodies at 4 °C for 30 min: anti-CD3 Alexa Fluor 647, anti-CD4 Pacific Blue, anti-CD8 RPE, and anti-WC1 FITC. The cells were suspended in 300 µL of PBS containing 2% fetal bovine serum. The cells were acquired and analyzed using a FACSCelesta flow cytometer (BD Biosciences, CA, USA) and FlowJo v10.10 Software. All antibodies were obtained from Bio-Rad Laboratories Inc., CA, UK. Data were analyzed using the gating strategy shown in Figure 1.

### 2.6. RNA Isolation and Quantitative Real-Time PCR

Total RNA was extracted from PBMCs using the Nucleospin RNA kit (Macherey-Nagel, Düren, Germany), and complementary DNA was synthesized using the ReverTra Ace qPCR RT Master Mix (TOYOBO, Osaka, Japan) according to the manufacturer’s instructions. Quantitative real-time PCR (qRT-PCR) was performed using Maxima Sybr Green qPCR Mastermix (Thermo Inc., MA, USA), and gene expression levels were measured using a QuantStudio™3 Real-Time PCR system (Applied Biosystems). Table 1 contains the primer sequences.

### 2.7. Malondialdehyde (MDA) Assessment

Serum MDA levels were determined using the thiobarbituric acid reactive substance (TBARS) method, as described previously [26]. Briefly, 50 µL of serum was mixed with 200 µL of 0.9% NaCl (QRëC™, Auckland, New Zealand) and 100 µL of 0.12 M thiobarbituric acid (Fluka, Steinheim, Germany). Subsequently, 500 µL of 10% trichloroacetic acid (QRëC™, Auckland, New Zealand) was added. After heating in a boiling water bath for 30 min, 1000 µL of distilled water was added, and the mixture was cooled, vortexed, and centrifuged for 10 min at 1100× *g*. The supernatant was measured at 532 nm using a microplate reader (Tecan Infinite^®^200, Männedorf, Switzerland). A standard calibration curve was generated using 1,1,3,3-tetraethoxypropane (Aldrich, Steinheim, Germany).

### 2.8. Nitric Oxide Assessment

Serum nitric oxide levels were measured using the Griess assay, as described previously [26]. Briefly, 100 µL of serum was mixed with 100 µL of Griess solution (1% sulfanilamide (DC Fine Chemicals, Terrassa, Spain)), and 0.1% N-(1-naphthyl) ethylenediamine dihydrochloride (PanReac AppliChem, Darmstadt, Germany) in 2.5% phosphoric acid (QRëC™, Auckland, New Zealand) and incubated at room temperature for 15 min. The absorbance was measured at 540 nm, and the results were compared with a standard curve.

### 2.9. Total Antioxidant Capacity Assessment

The total antioxidant capacity (TAC) of serum was measured by means of the ferric reducing antioxidant power (FRAP) method, as indicated by Benzie and Strain [27]. Fresh FRAP solution was prepared by dissolving 20 mmol/L FeCl_3_ (PanReac AppliChem, Darmstadt, Germany), 10 mmol/L 2,4,6-tri-pyridyl-s-triazine solution (Aldrich, St. Louis, MO, USA) in 40 mmol/L HCl (QRëC™, Auckland, New Zealand), and 0.3 mol/L acetate buffer (Ajax Finechem Pty. Ltd., Taren Point, New South Wales, Australia) (pH 3.6) in a 1:1:10 ratio. Subsequently, 20 µL of serum was reacted with 200 µL of the FRAP solution in a 96-well plate (Thermo Fisher Scientific, New Port, United Kingdom), and the optical density was measured using a Tecan Infinite^®^200 microplate reader at 593 nm. The result was calibrated with ferrous sulfate (Ajax Finechem Pty. Ltd., Taren Point, New South Wales, Australia). The result was expressed as mM Fe^2+^ per gram of the serum sample.

### 2.10. Statistical Analysis

Data were first tested for normal distribution before proceeding. One-way analysis of variance (ANOVA) was used to study the separate effects of time and castration techniques on cytokine gene expression, T lymphocyte subpopulations, and biochemical parameters. The combined effects of castration methods (Burdizzo and surgical techniques) and time points (before castration and 3, 6, 24, and 48 h post-castration) were analyzed using two-way ANOVA, with mean separations conducted using DMRT at a significance level of *p* < 0.05.

## 3. Results

### 3.1. Effect of Castration on Cytokine Gene Expression in PBMCs

The gene expression for the cytokine response of PBMCs was measured before castration and 3, 6, 24, and 48 h post-castration using qRT-PCR. In the Burdizzo castration group, the expression of IFN-γ at 6 h was significantly higher than before castration and 24 and 48 h post-castration (*p* < 0.05). In the surgical castration group, the IFN-γ showed no significant changes post-castration (*p* > 0.05). When comparing between groups, IFN-γ expression in the Burdizzo group was significantly higher than in the surgical group at both 3 and 6 h post-castration (*p* < 0.05).

In the Burdizzo castration group, the expression of TNF-α at 24 and 48 h post-castration was significantly higher than before castration and at 3 and 6 h post-castration (*p* < 0.05). In the surgical castration group, the TNF-α showed no significant changes post-castration (*p* > 0.05). Between groups, TNF-α expression was significantly higher in the Burdizzo castration group compared to the surgical castration group at both 24 and 48 h post-castration (*p* < 0.05).

**Figure 2:**
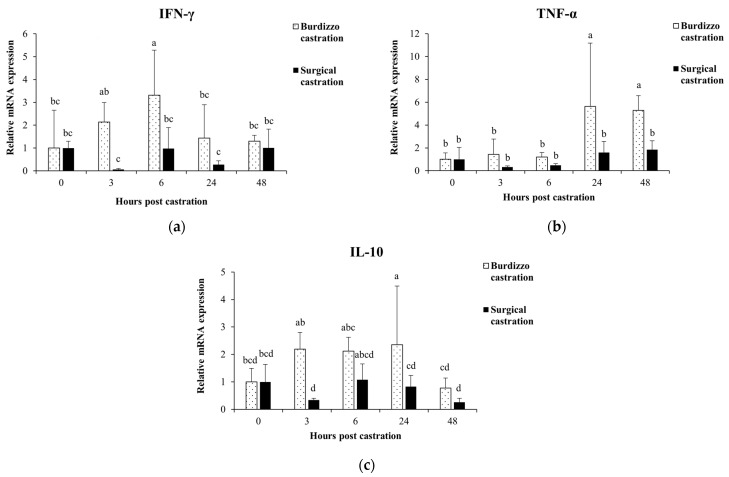
Effect of castration methods (Burdizzo and surgical castration) and time points (before castration and 3, 6, 24, and 48 h post-castration) on the expression of the cytokines (**a**) IFN-γ, (**b**) TNF-α, and (**c**) IL-10 in PBMCs. The values with different letters are significantly different (*p* < 0.05).

In the Burdizzo castration group, the expression of IL-10 at 24 h post-castration was significantly higher than before castration and at 48 h post-castration (*p* < 0.05). In the surgical castration group, the IL-10 showed no significant changes post-castration (*p* > 0.05). Between groups, IL-10 expression was significantly higher in the Burdizzo castration group compared to the surgical castration group at both 3 and 24 h post-castration (*p* < 0.05) (Figure 2).

### 3.2. Effect of Castration on T Lymphocyte Subsets in the PBMCs

The T lymphocyte subpopulations, including CD3^+^CD4^+^, CD3^+^CD8^+^, and CD3^+^WC1^+^, in the PBMCs of the cattle undergoing Burdizzo and surgical castration were analyzed. In the Burdizzo castration group, the percentage of CD3^+^CD4^+^ T lymphocytes at 24 h was significantly higher than at 3 and 48 h (*p* < 0.05), and the percentage of CD3^+^CD4^+^ T cells at 6 h was significantly higher than at 48 h post-castration (*p* < 0.05). In the surgical castration group, the percentage of CD3^+^CD4^+^ T cells at 6 and 24 h was significantly higher than at 3 and 48 h post-castration (*p* < 0.05).

The percentage of CD3^+^CD8^+^ T lymphocytes in the Burdizzo castration group at 48 h was significantly lower than at 6 and 24 h post-castration (*p* < 0.05). In the surgical castration group, the percentage of CD3^+^CD8^+^ T lymphocytes at 48 h was significantly lower than before castration and at 6 and 24 h post-castration (*p* < 0.05). The percentage of CD3^+^CD8^+^ T lymphocytes at 24 h was significantly higher than at 3 h post-surgical castration (*p* < 0.05).

**Figure 3:**
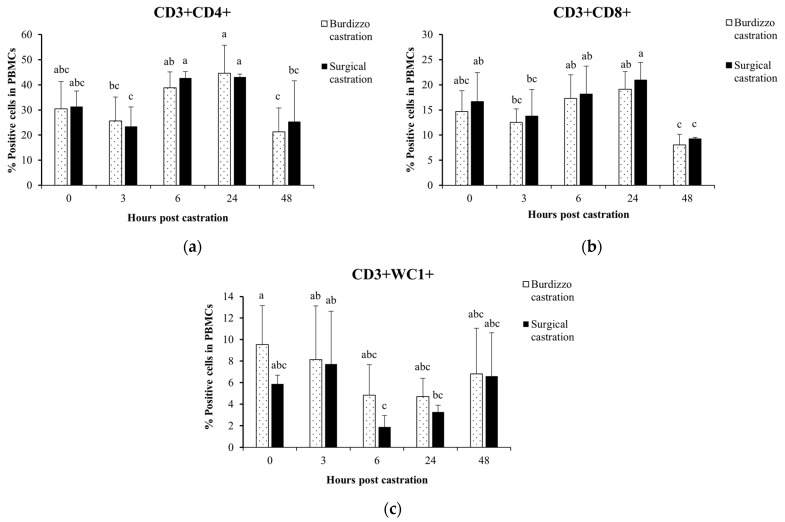
Effect of castration methods (Burdizzo and surgical castration) and time points (before castration and 3, 6, 24, and 48 h post-castration) on subpopulations of the T lymphocytes (**a**) CD3^+^CD4^+^, (**b**) CD3^+^CD8^+^, and (**c**) CD3^+^WC1^+^ in the PBMCs. The values with different letters are significantly different (*p* < 0.05).

In the Burdizzo castration group, there were no significant changes in the percentage of CD3^+^WC1^+^ T lymphocytes post-castration (*p* > 0.05). In the surgical castration group, the percentage of CD3^+^WC1^+^ T lymphocytes at 6 h was significantly lower than at 3 h post-castration (*p* < 0.05) (Figure 3).

### 3.3. Effect of Castration on MDA, Total Antioxidant Capacity and NO in Calves

**Figure 4:**
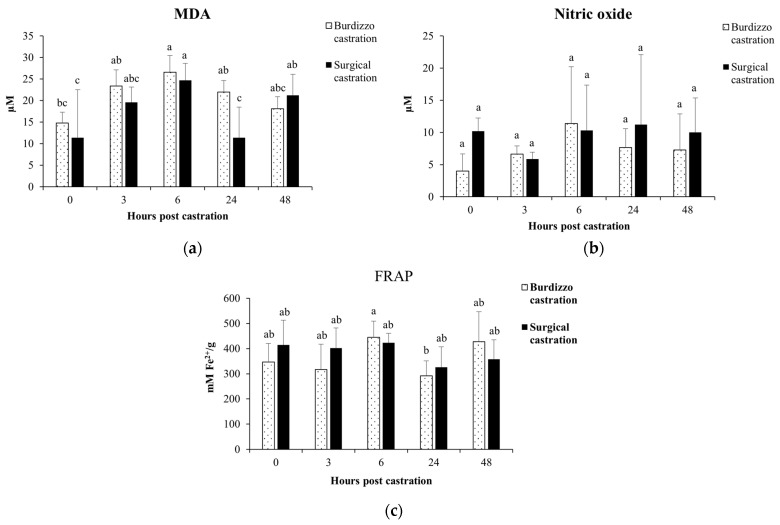
Effect of castration methods (Burdizzo castration and surgical castration) and time points (before castration and 3, 6, 24, and 48 h post-castration) on (**a**) malondialdehyde; (**b**) nitric oxide; and (**c**) total antioxidant capacity levels in calves. The values with different letters are significantly different (*p* < 0.05).

In the Burdizzo castration group, the MDA level at 6 h post-castration was significantly increased (*p* < 0.05). In the surgical castration group, the MDA level at 6 and 48 h post-castration was significantly increased (*p* < 0.05). Additionally, the MDA level at 24 h post-Burdizzo castration was significantly higher than at 24 h post-surgical castration (*p* < 0.05). In both the Burdizzo and surgical castration groups, there were no significant changes in NO levels post-castration (*p* > 0.05). The total antioxidant capacity at 6 h post-Burdizzo castration was significantly higher than at 24 h post-Burdizzo castration (*p* < 0.05). In the surgical castration group, there were no significant changes in the total antioxidant capacity post-castration (*p* > 0.05) (Figure 4).

## 4. Discussion

Castration is a widely used technique in livestock management with the purposes of reducing problems related to aggressive behavior and undesirable reproductive behavior and improving meat quality by reducing the incidence of dark meat and high-pH meat, which in turn increases the productivity of beef cattle farmers [28]. However, castration induces serious physiological and pathological responses, particularly inflammation and immune system alterations, leading to oxidative stress and alterations in antioxidant and nitric oxide levels in animals [20,21]. Therefore, studying these changes is important for improving castration practices in beef cattle to protect welfare while increasing farm productivity.

Surgical castration induces acute stress and inflammation, resulting in marked changes in immune and oxidative stress markers [10,24,29]. In this study, IFN-γ levels appeared to decrease within 3 h post-surgical castration and then returned close to baseline by 6 h, although these changes were not statistically significant. This two-step response indicates an early immune suppression following surgical castration injury and the release of cortisol, the body’s primary stress hormone. This response is consistent with the findings of Ertuğrul et al. [17], who found that IFN-γ levels fluctuate under surgical stress and return to normal levels once the immune system is in balance. On the other hand, in the Burdizzo castration group, the IFN-γ levels increased at 3 h, reached a peak at 6 h, and then decreased by 48 h post-castration, indicating the presence of acute inflammation caused by ischemia from the Burdizzo castration. This response corresponds to the findings that ischemia, resulting from banding or Burdizzo castration, subsequently triggers necrosis and inflammation. This acute inflammatory response is driven and maintained by the involvement of cytokines such as TNF-α, IL-1, IL-6, and IFN-γ [30]. IFN-γ plays a key role in the immune system, including control of postoperative wound infection [12]. This recovery period is crucial for promoting animal welfare and reduces the chance of postoperative infection.

An increased level of TNF-α, a primary pro-inflammatory cytokine, was not observed at the early time points of 3 and 6 h, but was observed at 24 and 48 h post-Burdizzo castration, indicating that castration causes a delayed increase in inflammatory cytokine response [17]. TNF-α is related to the degree of inflammation and tissue damage in a dose-dependent manner [31]. As an acute immune response mediator, the delayed escalation of TNF-α suggests that castration leads to extended tissue inflammation. This response corresponds to the finding that TNF-α is a marker of tissue injury that intensifies with continued healing [31]. The lower TNF-α level before castration and at 3 and 6 h post-castration compared to 24 and 48 h post-castration highlights a dual-phase inflammatory response, reflecting the study by Baldwin et al. [32] on temporal immune modulation in response to trauma.

Our study showed that IL-10 expression peaked at 24 h post-Burdizzo castration, with statistically significant differences compared to baseline and 48 h. This suggests a transient anti-inflammatory feedback response. This pattern aligns with studies by Warnock et al. [19] and Al-Qahtani et al. [33], which indicated that IL-10 serves as a feedback regulator to prevent chronic inflammation during recovery. In addition, studies in stressed and castrated Wistar rats showed that the IL-10 response was influenced by the level of stress. While stress increased both pro- and anti-inflammatory cytokines, castration primarily increased pro-inflammatory markers, with IL-10 levels significantly increasing only following stress and castration [34]. This response may play an important role in the restoration of the immune system after castration, avoiding uncontrolled inflammation and tissue damage and aiding in a smooth tissue healing process. The observed cytokine responses following castration may also be interpreted within the framework of Th1/Th2 immune regulation. IFN-γ is a key cytokine produced by Th1 cells, associated with cellular immunity, macrophage activation, and acute inflammation, whereas IL-10 is produced predominantly by Th2 cells and plays a key role in suppressing Th1 responses and limiting tissue damage [33]. The significant upregulation of IFN-γ in the Burdizzo castration group at 6 h suggests a transient Th1-dominant response triggered by ischemic injury. Conversely, the elevation of IL-10 at 24 h may reflect a compensatory activation of Th2-mediated aimed at restoring immune balance and preventing chronic inflammation. Previous studies have reported that surgical trauma or stress can transiently disturb the Th1/Th2 balance, initially favoring Th1 activation followed by a shift toward Th2 or immunosuppressive states during recovery [19,35]. These sequential changes in cytokine expression in our study support the idea of dynamic Th1/Th2 regulation following castration and highlight potential differences in immune resolution between castration techniques.

A previous study showed that MDA, a marker of oxidative stress, increased significantly at 6 h post-castration, indicating lipid peroxidation from ROS generation [23]. MDA is an important biomarker for assessing tissue damage after trauma or surgery. The decline in MDA levels by 24 h suggests that the body’s antioxidant mechanisms are working to reduce oxidative stress levels and restore balance. Meanwhile, surgical castration resulted in generally lower MDA levels than Burdizzo castration, with a temporary increase at 6 h followed by a decrease at 24 h. The comparatively lower MDA levels observed in the surgical castration group may indicate lesser oxidative stress, although these differences were not consistently statistically significant. This trend may suggest less ischemic injury and potentially shorter recovery periods [22].

The body’s antioxidant response following Burdizzo castration is reflected in the elevated FRAP levels at 6 h post-castration. Silvestrini et al. [36] reported that antioxidant systems can quickly respond to acute oxidative challenges to prevent excessive tissue damage. The rise in antioxidant capacity observed at 6 h post-Burdizzo castration may reflect an acute response to oxidative stress. Although statistical significance was limited to specific time points, the trend suggests the body’s early attempt to restore redox balance.

This study showed that CD3^+^CD4^+^ and CD3^+^CD8^+^ T lymphocyte levels increased at 6 and 24 h post-surgical castration, reflecting the immune system’s response to tissue injury. The recovery of the peripheral lymphocyte starts at 24 h after surgery, following an initial decrease in circulating lymphocytes caused by injured trauma that occurs within a few hours [35]. It has been demonstrated that T helper cells can particularly affect wound healing during inflammation by invading lymphocytes. CD4 and CD8 Th cells may play distinct regulatory functions during tissue regeneration. Our result is consistent with the study by Yoo et al. [18], which found that T-cell responses were elevated due to trauma from castration. This finding suggests that elevated CD3^+^CD4^+^ lymphocyte counts during the early post-castration period could be crucial for tissue regeneration and healing as well as a useful technique to enhance surgical results. In contrast, Burdizzo castration is a bloodless castration technique that involves compression of the spermatic cord and blood vessels and blocking the blood supply to the testicles without skin incision, resulting in a delayed immune response compared to surgical castration [6]. CD3^+^CD4^+^ levels increased at 24 h post-castration, indicating a gradual immune response, possibly due to less immediate injury. *In vitro*, γδ T cells have been shown to suppress antigen-specific and nonspecific CD4^+^ and CD8^+^ T cell proliferation and function [37]. Our data showed that peripheral CD3^+^WC1^+^ lymphocytes decreased at 6 and 24 h post-castration. Moreover, considering the correlation between the increased levels of CD3^+^CD4^+^ lymphocytes and reduced levels of CD3^+^WC1^+^ lymphocytes at 6 and 24 h post-castration, these occurrences indicated that bovine γδ T cells highlight the immune system’s adjustment in the early phase of inflammation [32].

Interestingly, the levels of IFN-γ, TNF-α, and IL-10 were higher in the Burdizzo group than in the surgical group. Burdizzo castration can induce a robust immune response, possibly due to ischemic injury to the testicles from spermatic cord and blood vessel compression. This is consistent with the study by Pang et al. [38], who observed that ischemic trauma to the testicles can induce inflammation even with minor tissue injury in the affected area. Up to 48 h after castration, the acute phase was the main focus of this investigation. The recovery process and the effects of chronic stress might be better understood with additional research on the long-term inflammatory and immunological responses.

The physiological reactions of calves to the two castration methods were examined in this study. Indicators of oxidative stress, peripheral blood mononuclear cell populations, and inflammatory cytokines changed following both castrations. Notably, surgical castration induces a moderate immune response and oxidative stress levels, associated with less severe tissue injury. This finding is similar to the results by Roberts et al. [6] and Petherick et al. [39], which showed that both methods caused stress and that surgical castration might result in less pain and stress for the animals.

Despite the small study group size, the findings offer some initial understanding of the immunological response, oxidative stress, and inflammatory response after different castration techniques. To validate these results and increase the results’ generalizability, larger sample sizes are required for future research.

## 5. Conclusions

This study demonstrates that Burdizzo and surgical castration elicit different immunological and oxidative stress responses in beef calves during the acute post-castration period. Both methods induce changes in the levels of pro-inflammatory cytokines such as TNF-α and IFN-γ and oxidative stress indicators such as MDA. However, the bloodless Burdizzo method results in a more intense and prolonged inflammatory process and increased oxidative stress, while surgical castration elicits a more moderate immune and oxidative response. However, this study did not assess clinical outcomes relevant to castration. Therefore, any potential welfare advantages suggested by the cytokine and oxidative markers must be balanced against these procedural risks. Future studies with larger sample sizes and longer study periods are required to fully assess the physiological and clinical effects of various castration techniques. These studies will support evidence-based management strategies that maximize production and animal welfare in cattle farms.

## Figures and Tables

**Table 1:** Primers used for qRT-PCR.

Gene	Forward Primer	Reverse Primer
IFNG	5′- TTGAATGGCAGCTCTGAGAAAC	5′- TCTCTTCCGCTTTCTGAGGTTAGA
IL-10	5′- AAGGTGAAGAGAGTCTTCAGTGAGC	5′- TGCATCTTCGTTGTCATGTAGG
TNFA	5′- TGACGGGCTTTACCTCATCT	5′- TGATGGCAGACAGGATGTTG
GAPDH	5′- GGC GTG AAC CAC GAG AAG TAT AA	5′- CCC TCC ACG ATG CCA AAG T

## Data Availability

The data presented in this study are available in this article.
